# The Inhibitory Effect of Protamine on Platelets is Attenuated by Heparin without Inducing Thrombocytopenia in Rodents

**DOI:** 10.3390/md17090539

**Published:** 2019-09-17

**Authors:** Joanna Miklosz, Bartlomiej Kalaska, Kamil Kaminski, Malgorzata Rusak, Krzysztof Szczubialka, Maria Nowakowska, Dariusz Pawlak, Andrzej Mogielnicki

**Affiliations:** 1 Department of Pharmacodynamics, Medical University of Bialystok, 15-222 Bialystok, Poland; 2 Department of Physical Chemistry, Faculty of Chemistry, Jagiellonian University, 30-387 Krakow, Poland; 3 Department of Haematological Diagnostics, Medical University of Bialystok, 15-269 Bialystok, Poland

**Keywords:** animal models, heparin, platelets, protamine, thrombocytopenia, thrombosis

## Abstract

Protamine sulfate (PS) is a polycationic protein drug obtained from the sperm of fish, and is used to reverse the anticoagulant effect of unfractionated heparin (UFH). However, the interactions between PS, UFH, and platelets are still not clear. We measured the platelet numbers and collagen-induced aggregation, P-selectin, platelet factor 4, β-thromboglobulin, prostacyclin metabolite, D-dimers, activated partial thromboplastin time, prothrombin time, anti-factor Xa, fibrinogen, thrombus weight and megakaryocytopoiesis in blood collected from mice and rats in different time points.. All of the groups were treated intravenously with vehicle, UFH, PS, or UFH with PS. We found a short-term antiplatelet activity of PS in mice and rats, and long-term platelet-independent antithrombotic activity in rats with electrically-induced thrombosis. The antiplatelet and antithrombotic potential of PS may contribute to bleeding risk in PS-overdosed patients. The inhibitory effect of PS on the platelets was attenuated by UFH without inducing thrombocytopenia. Treatment with UFH and PS did not affect the formation, number, or activation of platelets, or the thrombosis development in rodents.

## 1. Introduction

Protamine sulfate (PS), an alkaline protein consisting mainly of arginine, stabilizes DNA during spermatogenesis. PS is used in medicine to reverse the anticoagulant activity of anionic unfractionated heparin (UFH), and to stabilize neutral protamine Hagedorn (NPH) insulin [1]. Commercially available PS is currently derived from the sperm nuclei of chum salmon fish, which were traditionally caught at the north-eastern coast of the Japanese island of Honshu. The salmon fishing areas were moved north of the Hokkaido island after the Japan earthquake and tsunami of 2011. The differences among the individual fish acquired from the separate geographical populations resulted in a heterogeneity of PS and its action. PS also may induce an anaphylactic reaction in patients receiving NPH insulin or those with a fish allergy, which is probably related to its animal origin. Despite the above, PS is still in use as it is a life-saving drug [1]. The postoperative PS infusion minimizes bleeding after UFH administration [2]. However, the risk still exists and may increase by both the release of UFH from complexes with PS [3], or with the additional doses of PS [4]. 

The anticoagulant properties of PS are related to its interaction with platelets, coagulation factors, and fibrinolysis [5]. Under physiological conditions, platelets freely circulate in the blood vessels, and they are protected from activation by the healthy endothelium and its mediators, such as nitric oxide (NO) and prostacyclin. The activation of platelets is a crucial step in arterial thrombosis development. Adhesive glycoprotein receptors Ib/IX, Ia/IIa, VI, and IIb/IIIa interact with von Willebrand factor (vWF) and collagen, among others, which capture the platelets and induce activation signals. The activated platelets secrete agonists from dense- and α-granules, such as adenosine diphosphate, platelet factor 4 (PF4), or β-thromboglobulin (βTG), leading to further activation. Platelets serve as attachment sites for coagulation factors, and as a source of those factors and other molecules, such as polyphosphates (PolyP) and prothrombin. The initiated coagulation cascade and aggregated platelets contribute to fibrin formation, which stabilizes the thrombus [6]. Direct exposure to PS may reduce the platelet activity and aggregation, and induce thrombocytopenia. Transient thrombocytopenia (5–60 min) was reported during UFH neutralization in female Sprague-Dawley rats [7], dogs [8], goats [9], and humans [10]. Previous in-vitro studies have suggested that PS may adhere to the negatively charged platelet membrane, and thus even induce platelet aggregation by forming bridges between adjacent platelets, leading to thrombocytopenia [11]. It also interferes with glycoprotein Ib (GPIb) [12], which plays a critical role in mediating platelet clearance [13]. PS complexed with UFH could alter the platelet behavior and form complexes with them, which accumulate in the pulmonary and hepatic circulation [7,8,9,10,14,15,16,17,18,19]. Some authors postulated that UFH and PS change blood cells through the classical pathway of complement activation, which may result in transient thrombocytopenia [20]. There are many studies with often contradictory results on the short-term antiplatelet action of PS, but there is a lack of information on the potential long-term platelets’ response. The clinical observation is scarce and can hardly be attributed to PS.

Recently, it was proposed that multimolecular complexes of UFH and PS may induce the production of platelet-activating immunoglobulin G (IgG) antibodies, which are responsible for severe thrombocytopenia and thromboembolic complications [21,22,23,24,25]. This phenomenon has been observed in the post-operative period, and more frequently in immunized patients with NPH insulin [21,22,23,24,26,27]. The Fc domains of the IgG antibody within the immune PS and UFH complex may bind to the platelet Fcγ receptor type IIa, and activate platelets, leading to their aggregation and clearance, but other Fc-independent mechanisms could also be taken into account [12,21,28,29]. However, a significant part of the current data was obtained from patients taking more than one drug and undergoing invasive cardiovascular procedures, which can mask the real effects of PS. The discrepancies may also be related to the different times of platelet responses or the presence of antibodies.

The gaps in the potential hemostatic complications related to UFH neutralization by PS, and the unclear platelet response to PS encouraged us to explore, in more detail, the interaction of UFH and PS with platelets and thrombosis, with the use of relevant animal in-vivo models in a time-dependent manner. In the present study, we investigated the number of platelets and the various markers of platelet activation in mice and rats from three minutes up until five weeks, from single or repeated injections of PS alone, or PS together with UHF. We also estimated the thrombopoiesis, as well as the mechanisms involved in the thrombotic and hemorrhagic complications of drugs.

## 2. Results

### 2.1. The Effect of UFH and PS on the Number of Platelets and Their Function in Mice and Rats

We decided to choose a therapeutic dose of UFH (150 U/kg) that extended the activated partial thromboplastin time (aPTT) by almost three times and the bleeding time by half in rats [30]. The PS dose was determined based on the clinical practice, finding that 1 mg of PS neutralizes 100 U of UFH. 

**Figure 1:**
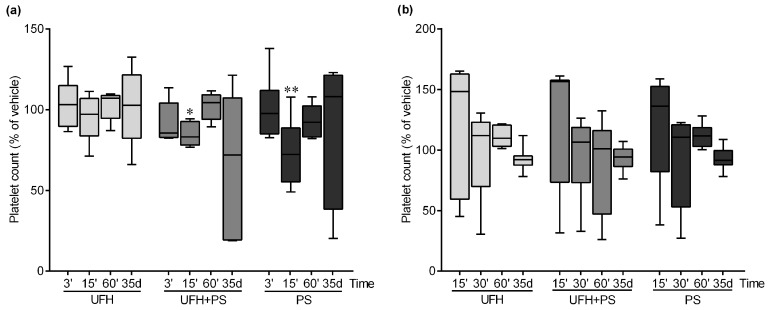
Platelet number in (**a**) mice (*n* = 5–8) and (**b**) rats (*n* = 5–10) at 3, 15, 30, and 60 minutes, and on the 35th day after the administration of unfractionated heparin (UFH) and protamine sulfate (PS). The results are expressed as a percentage of the control samples, and are shown as a median (line) with the interquartile range (box), and maximum and minimum values (whiskers). The number of platelets in the vehicle-treated groups was 617 (565–675) at 3 and 15 minutes, 705 (670–752) at 60 minutes, and 427 (326–467) on the 35th day in the mice. In the rats, the control values were 446 (176–754), 725 (372–746), 668 (196–781), and 637 (494–660) at 15, 30, and 60 minutes, and on the 35th day, respectively. * *p* < 0.05; ** *p* < 0.01 vs. vehicle group; Kruskal–Wallis analysis of variance (ANOVA) with Dunn’s post-hoc test.

PS alone slightly decreased the platelet count at 15 min in the mice, while we observed a similar but even smaller decrease in the platelet count in a group treated with UFH and PS. The number of platelets returned to a normal level at the 60 min in both groups (Figure 1a). 

There was no statistical difference in platelet count in the mice treated once a week with UFH and PS, or PS alone for 35 days, but we noted a drop in the number of platelets, to below 50%, in three out of the seven mice treated with UFH and PS at the end of the experiment (Figure 1a). In the rats, we did not observe any changes in the platelet count during the whole experiment (Figure 1b). 

**Figure 2:**
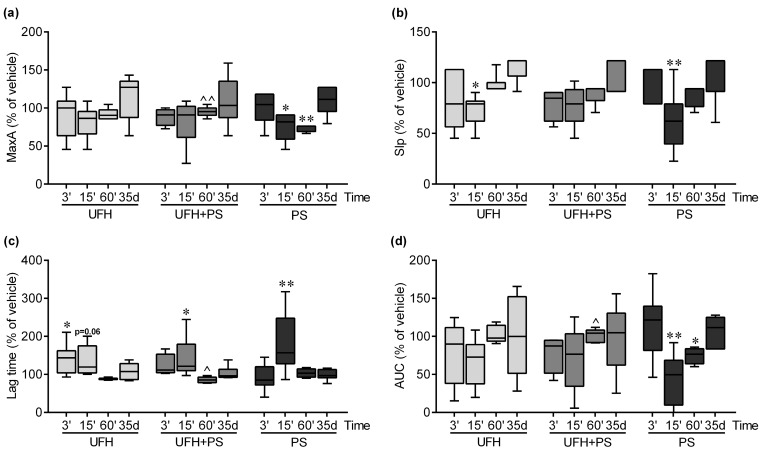
Platelet aggregation results in mice at 3, 15, and 60 minutes (*n* = 4–7), and on the 35th day (*n* = 5–7) after unfractionated heparin (UFH) and protamine sulfate (PS) administration. (**a**) Collagen-induced platelet aggregation expressed as the maximal extension (MaxA), (**b**) the slope of platelet aggregation (Slp), (**c**) lag time, and (**d**) the area under the curve (AUC). The results are expressed as a percentage of the control samples, and are shown as the median (line) with the interquartile range (box), and maximum and minimum values (whiskers). The control values at 3 and 15 minutes were 11.0 (10.0–13.0), 9.0 (6.0–10.0), 115.0 (104.0–174.0), and 31.3 (21.1–40.4); at 60 minutes they were 10.5 (10.0–11.0), 4.0 (4.0–5.0), 137.0 (112.0–151.0), and 25.5 (21.4–30.6); and on the 35th day, they were 6.0 (4.0–8.0), 3.0 (3.0–4.0), 190.0 (150.0–270.0), and 11.6 (3.8–15.7), for MaxA, Slp, lag time, and AUC, respectively. * *p* < 0.05; ** *p* < 0.01 vs. vehicle; ^ *p* < 0.05; ^^ *p* < 0.01 vs. PS group; Kruskal–Wallis ANOVA with Dunn’s post-hoc test.

**Figure 3:**
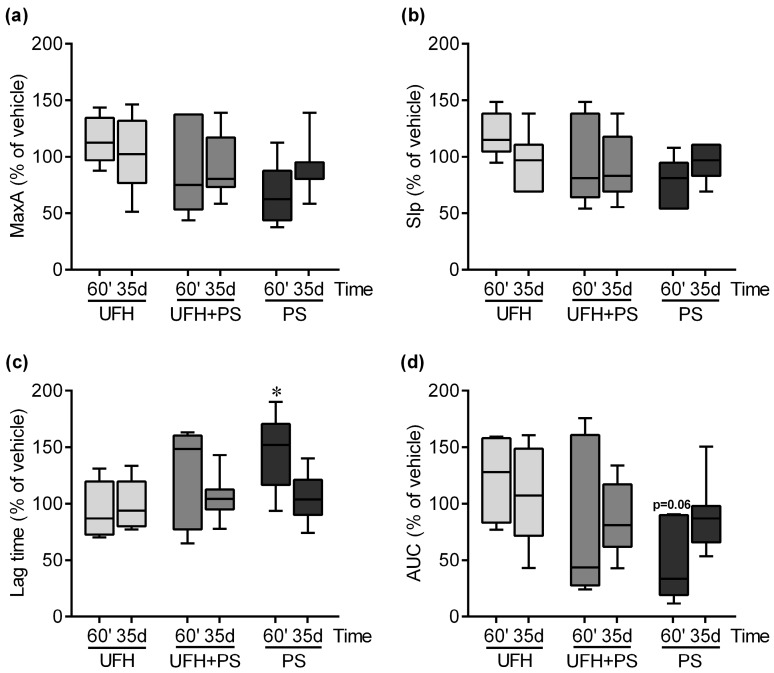
Platelet aggregation results in rats at 60 min (*n* = 5–7) and on the 35th day (*n* = 9–10) after unfractionated heparin (UFH) and protamine sulfate (PS) administration. (**a**) Collagen-induced platelet aggregation expressed as the maximal extension (MaxA), (**b**) the slope of platelet aggregation (Slp), (**c**) lag time, and (**d**) area under the curve (AUC). The results are expressed as a percentage of the control samples and are shown as the median (line) with the interquartile range (box), and maximum and minimum values (whiskers). The control values at 60 min were 8.5 (6.0–10.5), 4.0 (3.0–4.5), 156.0 (102.5–199.0), and 20.7 (9.7–25.2), and on the 35th day were 7.0 (3.5–9.0), 4.0 (2.0–5.0), 133.5 (85.0–220.0), and 17.4 (5.4–27.3) for MaxA, Slp, lag time, and AUC, respectively. * *p* < 0.05 vs. vehicle group; Kruskal–Wallis ANOVA with Dunn’s post-hoc test.

PS administered alone significantly inhibited the platelet aggregation in the mice at 15 and 60 min (Figure 2), and in the rats at 60 min (Figure 3). UFH attenuated the inhibitory effect of PS on the platelets (Figure 2). The UFH and PS treatment only slightly delayed collagen-induced platelet aggregation 15 min after a single injection into the mice (Figure 2). We observed no changes in the platelet aggregation after 35 days in the mice (Figure 2) and rats (Figure 3).

**Figure 4:**
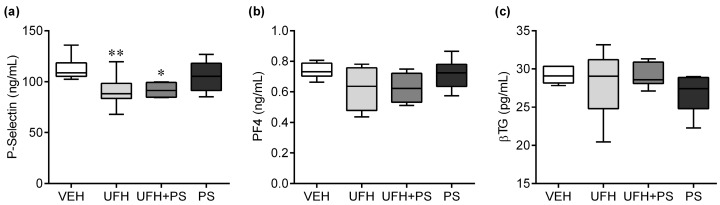
Effects of unfractionated heparin (UFH) and protamine sulfate (PS) on (**a**) P-selectin, (**b**) platelet factor 4 (PF4), and (**c**) β-thromboglobulin (βTG) concentration in mice (*n* = 6–8) on the 35th day of the experiment. The results are shown as the median (line) with the interquartile range (box), and maximum and minimum values (whiskers). * *p* < 0.05; ** *p* < 0.01 vs. vehicle group; Kruskal–Wallis ANOVA with Dunn’s post-hoc test. VEH—vehicle.

We observed a significant reduction in the P-selectin concentration (Figure 4a), and no changes in the PF4 (Figure 4b) and βTG concentration (Figure 4c) in the mice treated repeatedly (once a week) with UFH alone, or together with PS, for 35 days. 

### 2.2. The Effect of UFH and PS on Thrombosis and Coagulation Parameters in Mice and Rats

**Figure 5:**
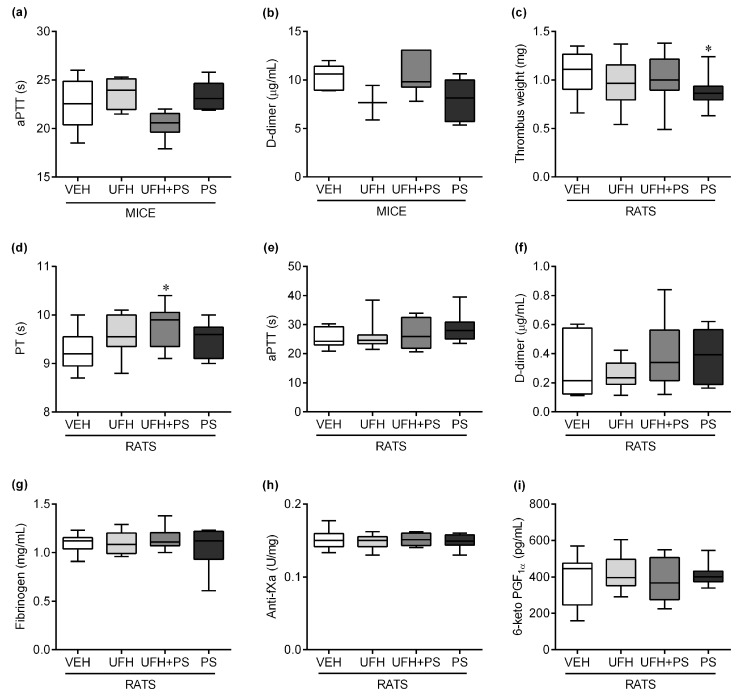
Effects of unfractionated heparin (UFH) and protamine sulfate (PS) on the (**a**) activated partial thromboplastin time (aPTT) and (**b**) D-dimer concentration in mice (*n* = 3–7), and (**c**) thrombus weight, (**d**) prothrombin time (PT), (**e**) activated partial thromboplastin time (aPTT), (**f**) D-dimer concentration, (**g**) fibrinogen concentration, (**h**) anti-factor Xa (anti-fXa) activity, and (**i**) 6-keto PGF_1α_ concentration in rats (*n* = 9–10) on the 35th day of the experiment. The results are shown as the median (line) with the interquartile range (box), and maximum and minimum values (whiskers). * *p* < 0.05; ** *p* < 0.01; Kruskal–Wallis ANOVA with Dunn’s post-hoc test.

There was no change in the aPTT (Figure 5a) and D-dimer (Figure 5b) concentration in the mice. The PS significantly decreased the thrombus weight after repeated intravenous administration in the rats developing arterial thrombosis (0.86 (0.63–1.24) vs. 1.11 (0.66–1.35) mg in the vehicle group, Figure 5c). The UFH administered together with PS slightly prolonged the prothrombin time (PT; Figure 5d) in the rats on the 35th day of the experiment. There was no change in the aPTT (Figure 5e), D-dimer (Figure 5f) concentration, fibrinogen (Figure 5g) concentration, anti-factor Xa (anti-fXa, Figure 5h) activity, and 6-keto PGF_1α_ (Figure 5i) concentration in the rats. In general, except for the thrombus weight, all of the significant changes in the coagulation parameters did not exceed 5%.

### 2.3. The Effect of UFH and PS on Megakaryocytopoiesis in Mice

**Figure 6:**
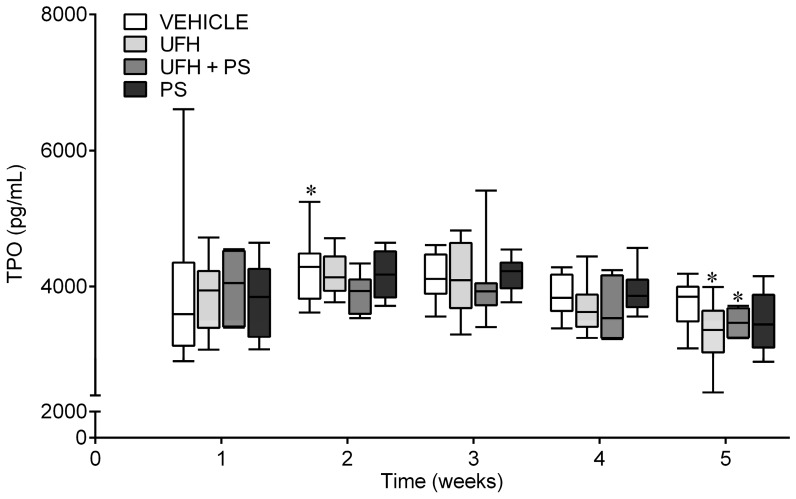
The thrombopoietin level (TPO) during the repeated administration of unfractionated heparin (UFH) and protamine sulfate (PS) in mice (*n* = 6–8). The serum TPO concentration was measured by specific enzyme-linked immunosorbent assay (ELISA). The results are shown as the median (line) with the interquartile range (box), and maximum and minimum values (whiskers). * *p* < 0.05 vs. the first week within the same group; Friedman ANOVA with Dunn’s post-hoc test.

**Figure 7:**
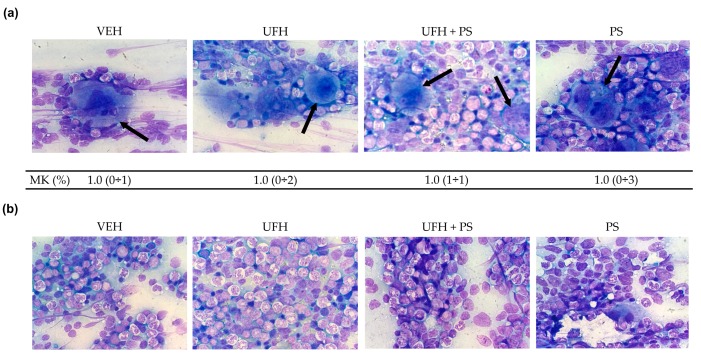
The morphology of megakaryocytes (MK), and their number per 100-cell count for bone marrow (%), shown as the median with range. (**a**) The arrows in the top panel indicate MK, and (**b**) the bottom panel represent the remaining bone marrow cell lines at a 1000-fold magnification in bone marrow smears from mice (*n* = 8) treated repeatedly with unfractionated heparin (UFH) and protamine sulfate (PS). May–Grünwald–Giemsa stain (Merck) following the standard protocol. Microscope: Olympus CH 30; objectives: DPlanC 100, 1.25 oil, 160/0.17; DPlanC 40, 0.65, 160/0.17; camera: Digital Sight DS.-Fi1 Nikon.

The thrombopoietin (TPO) levels decreased in the mice treated with UFH alone, or with UFH with PS, after five weeks, compared with the levels measured one week after the first injection; but no changes were observed in comparison to the vehicle-treated group (Figure 6). The analysis of the hematopoietic composition of the bone marrow showed a similar percentage of megakaryocytes in each preparation from the mice treated repeatedly with UFH and PS alone or together (Figure 7a). No morphological differences in any of the bone marrow cells were observed between the control and test groups (Figure 7a,b). The median counts of the total red and white blood cells, monocytes, and lymphocytes are shown in Table S1. We reported significant differences between the vehicle and PS groups with regard to the percentage of polychromatic erythroblasts, and the total erythroid cells (E) to total myeloid cells (M) ratio (M/E). The mice repeatedly exposed to UFH and PS showed a significantly lower percentage of basophilic erythroblasts, without affecting the M/E ratio. We observed a relatively high percentage of metamyelocytes in the UFH and PS treated group, which could be attributed to PS itself. The mice in the UFH group showed a reduced percentage of promyelocytes and lymphocytes, of which the latter cell category was found to be significantly lower in the group treated with UFH and PS. We did not find the platelet clusters in any of the preparations, but all of them had damaged cells and fibers as a result of the smear technique.

### 2.4. The Effect of UFH and PS on the Blood Count in the Mice and Rats

UFH and PS mainly changed the parameters characterizing the red blood cells shortly after concomitant administration into both rodents. However, the decrease in the red blood cell number, hemoglobin level, haematocrit, mean corpuscular haemoglobin, and mean corpuscular haemoglobin concentration, and the increase in the mean corpuscular volume were slight and in the normal range (below 8% in comparison to control group). PS alone significantly decreased the number of erythrocytes, the hemoglobin level, and hematocrit in the rats (Table S2), but not in the mice (Table S3), 60 min after injection. UFH alone reduced the number of red blood cells, while increasing their mean volume after 3 min in the mice (Table S3). Other than a 3.9% decrease in the mean corpuscular haemoglobin concentration after the repeated administration of UFH, and a 5.5% decrease in the haemoglobin level after the repeated administration of UFH with PS, we observed no changes in the blood count on day 35 of the experiment, in comparison to the vehicle-treated mice and rats (Tables S2 and S3).

## 3. Discussion

In our present study, we focused on the marine-origin drug, protamine sulfate, and its hemostatic complications during UFH neutralization, which presents an unresolved problem, with contradictory results published so far. We studied the effects of PS alone, or together with UFH, on the number of platelets and their function in rodents, from 3 min to 35 days after the drug administration. We also investigated the mechanisms of this interaction by measuring the activation markers of the platelets and coagulation, as well as the involvement of the megakaryocytopoiesis. We found an inhibitory effect of PS on platelet aggregation without inducing thrombocytopenia, which was attenuated by the concomitant administration of UFH in the mice. When injected once a week for five weeks, PS slightly inhibited the arterial thrombosis development in rats, without changing the platelet activity or their number, the clotting times, and the D-dimers levels in both rodents. The PS administered together with UFH in the therapeutic doses into the mice and rats did not induce significant thrombocytopenia, the activation of platelets, arterial thrombosis development and abnormalities in megakaryocytopoiesis, or platelet formation. 

We observed previously that PS slightly decreased the thrombus weight and platelet aggregation, and increased the tail bleeding time without changing the aPTT in rats during a 1-h experiment [30]. In the present study, PS inhibited the collagen-induced platelet aggregation. However, the effect appeared 15 min after administration, and lasted for 1 h. It seems that the antithrombotic effect is species-independent, because it occurred not only in the mice, but also in the rats. We did not observe any significant changes in the platelet numbers in the mice and rats treated with PS alone or with UFH, except for a slight trend to decrease at 15 min. Generally, in the studies reporting thrombocytopenia, the doses of PS were higher compared with our experiment. This effect could depend on the doses and ratio, strain, sex, or species. The mechanism underlying the antiplatelet effect of PS is multifactorial, and may include the inhibition of platelet aggregation [4,31], sensitivity [32], GPIb–vWF activity [12], P-selectin expression [33], thrombin generation [34], and the release of intracellularly stored adenosine diphosphate and PF4 [35]. However, most evidence comes from in vitro studies, which did not take into account the involvement of the endothelial response [5]. In one of the studies, PS inhibited the collagen-induced activation of the platelets exposed to shear stress [36]. PS consists of L-arginine, which is the physiological precursor of strong antiplatelet agent, NO, with an extremely short half-life [37]. The release of L-arginine into circulation could play a primary role in the antiplatelet effect. The polycationic structure of PS and its nonspecific interactions with various hemostatic elements can enhance this activity, especially considering that we observed no effect when PS was complexed with UFH. Possibly by the electrostatic binding of GPIb, PS impairs the GPIb–vWF activity and the adhesion of platelets to collagen. The GPIb–vWF interaction is mandatory for normal hemostasis, and its impairment leads to a reduced platelet aggregation and risk of bleeding [12]. Interestingly, it seems that UFH, a typical anticoagulant drug, attenuated the inhibitory effect of PS on platelets. Despotis et al. reported that lower doses of PS in relation to the UFH dose reduced the blood consumption, as a result of the better preservation of the coagulation system, including platelet function [38]. The mechanism by which UFH attenuates the inhibitory effect of PS on platelets may be the same as the mechanism by which PS is the antidote for the UFH anticoagulant effect. We have previously clearly shown that PS binds 100% of UFH, which reverses at least the anticoagulant effect of UFH [39]. There are also studies confirming that the well-known pulmonary toxicity of PS is weakened by UFH. The polycations damage the pulmonary vascular beds by the neutralization of the anionic endothelial surface, which leads to an increased vascular permeability. The negatively-charged UFH binds and removes the circulating PS from the bloodstream, or it is possible that UFH counters the effect of the positively-charged PS molecules [40,41,42]. Perhaps UFH could be therapeutic in some cases of life-threatening PS reactions, and the administration of UFH first should be considered. The data from patients after cardiopulmonary bypass (CPB) indicated that UFH dose-based PS administration resulted in its overdose and in the impairment of the coagulation system [43]. An approximately 1.4:1 PS to UFH ratio significantly prolonged the clotting time in the thromboelastometry [43]. PS management based on the UFH concentration avoids haemorrhagic complications after cardiac surgery when compared with the conventional approach [43,44]. In general, the PS dose to UFH should not exceed 1:1, in order to avoid bleeding complications. The serious cardiovascular side effects of PS led to a search for alternative replacers. Universal heparin reversal agents (UHRA), low molecular weight protamine, Dex40-GTMAC3, and heparin binding copolymers are all in the preclinical stage; aripazine is already in the clinical phase, whereas andexanet alfa is registered for the reversal of oral direct Xa inhibitors [37,45,46,47,48]. 

Recently, it was suggested that the long-term response of platelets to UFH and PS complexes can be different, especially in patients who have had contact with PS in the past. The administration of UFH and PS induces the formation of platelet-reactive anti-UFH/PS antibodies, which could be responsible for the increased risk of severe thrombocytopenia and thromboembolic complications in the postoperative period, especially after CPB [21,24,25]. We wanted to simulate this clinical scenario so as to study the long-term response of platelets to PS, or PS and UFH complexes. We previously found IgG antibodies in the blood of mice three weeks after the first injection of UFH and PS, achieving a maximal concentration in week five [39]. Therefore, we repeatedly administered, once a week for five weeks, PS or PS with UFH. In our experimental setting, all of the animals survived until day 35, and did not show any symptoms of thrombosis, and platelets activation. We did not observe any changes in the concentrations of platelet activation markers, βTG, PF4, and soluble P-selectin, for which raised levels may predict adverse cardiovascular events [49]. We found a decrease in the concentration of P-selectin in the mice treated with UFH and PS. As an even greater decrease was observed in the group treated with UFH alone, it could be as a result of PS-binding by UFH, which serves as a ligand for P-selectin [50]. We did not observe any changes in D-dimer concentration in the 35th day. The median platelet count did not significantly decrease in the animals exposed to UFH and PS, but in three out of the seven mice, the platelet count decreased by half. The prolonged immune-mediated thrombocytopenia may not result from the platelet depletion, but from their impaired formation [51,52]. Bakchoul et al. provided evidence that anti-UFH/PS antibodies can affect megakaryocytopoiesis in the presence of UFH and PS [22]. In our study, the UFH and PS group did not show marked signs of megakaryocyte disruption compared to the control group. The percentage of other bone marrow cells changed in the mice treated with PS together with UFH. However, the bone marrow morphology may reflect changes resulting from decreased body weight gain [53]. Also, we did not observe abnormalities in the thrombopoiesis. The TPO production was sufficient to induce the formation of new platelets in all of the groups for five weeks. We noticed a slight decrease in the TPO concentration in week five in the UFH alone, and in the UFH and PS treated group, compared to the first measurement. This effect and the prolonged PT might be indicators of liver damage, as we previously found cellular changes in the mice livers after the injection of UFH and PS [54,55].

In summary, we did not confirm that the PS or UFH and PS treatments carry a long-term risk of platelet activation and thrombocytopenia, at least in the mice. We repeated the same experiment in rats, as there were previously reported species differences in the platelets’ response to UFH and PS. We additionally electrically induced arterial thrombosis in the rats at the end of the experiment so as to study the long-term effect of PS on thrombosis development. A rat model also allowed us to collect more blood for measuring the coagulation markers in order to study the potential mechanisms of the anticoagulant effects of PS. Similar to the mice, we did not observe changes in the platelet number and platelets aggregation five weeks after the repeated administration of PS and UFH, and no effect on thrombosis. We only noticed about a 5% change of PT in the UFH and PS group. A trend toward an increase of PT in the UFH-treated rats suggests that UFH by itself could contribute to PT prolongation. There are reports that PS prolongs PT by the inhibition of the activity of factors V, VII, and X [56]. Thus, it also could be an additive effect of both drugs. A very low thrombosis ratio (2/591) was also noted in the patients reversed with PS during CPB [25], so it is possible we could not detect such a rare effect in normal animals. The platelet count or its recovery is impaired by hemorrhagic conditions or contact with artificial surfaces, a common situation during CPB in humans. The platelet responses may also depend on the different progression of cardiovascular disease or complex pharmacotherapy. The anatomy/physiology differences between rodents and humans could also be a reason, especially if we take into account the complex nature of the immune and hemostasis systems. In our animal study, we eliminated platelet-modifying factors so as to solely study the effect of UFH and PS. Perhaps the UFH and PS treatment can only slightly enhance the effect of other factors on the platelets during CPB. 

Interestingly, the repeated administration of PS slightly, but significantly, inhibited thrombosis, without any changes in the platelet aggregation and coagulation tests. Perhaps the once a week administration of short-acting PS inhibits the platelets temporarily and reversibly. Based on our results, we can rather exclude the mechanisms involving clotting factors or platelets as the primary reason for the long-term antithrombotic effect of PS. The results of other studies suggest that the enhancement of fibrinolysis could contribute to the long-term antithrombotic effect of PS. The thrombin generation in murine and human plasma was significantly reduced by PS in a dose-dependent manner [56,57]. However, Ni Ainle et al. suggested that thrombin inhibition plays only a minor role in the PS anticoagulation effect [56]. PS significantly decreased the clot strength and enhanced fibrinolysis, which might occur through several possible mechanisms [58]. The down-regulation of thrombin generation decreases the activation of the thrombin-activatable fibrinolysis inhibitor [56,57]. Furthermore, PS inhibits both the tissue factor-dependent and direct thrombin generation, and then fibrinogen polymerization and the cross-linking of fibrin [58]. In the turbidimetric assays performed by Kalathottukaren et al., PS increased the clot turbidity. According to the authors, this phenomenon may result from the nonenzymatic polymerization or precipitation of fibrinogen, the incorporation of PS into fibrinogen or fibrin, or by impairing thrombin generation. They showed that PS is incorporated within the clot structure, which led to an abnormal clot architecture and enhancement fibrinolysis [46]. As mentioned before, the repeated releases of profibrinolytic and vasorelaxant NO by L-arginine from PS could also play a role here [59]. The second important endothelial mediator able to exert an antithrombotic activity is prostacyclin [60,61,62], but we found no changes in the plasma concentration of prostacyclin metabolite, 6-keto-PGF1α, in the rats receiving PS. The antithrombotic activity of the synthetic cationic macromolecules, such as UHRA, was explained by their binding to endogenous polyanionic molecules, PolyP, with a prothrombotic activity [63]. PolyP, through the influence on the coagulation cascade, enhances the thrombin generation. In addition, it incorporates into fibrin clots to stabilize them [63]. Perhaps PS, as a natural cationic macromolecule, could also have a similar ability. 

## 4. Materials and Methods 

### 4.1. Materials

We used trisodium citrate (≥99%), dipotassium ethylenediaminetetraacetic acid (K_2_EDTA, analytical grade), PS from salmon (grade X; Sigma-Aldrich, Darmstadt, Germany), UFH from bovine intestinal mucosa (Polfa Warszawa, Warsaw, Poland), isoflurane (Baxter Polska, Warsaw, Poland), pentobarbital, ketamine, xylazine (Biovet, Pulawy, Poland), phosphate buffered saline (Biomed Lublin, Lublin, Poland), aPTT, PT and fibrinogen reagents (Bio-Ksel, Grudziadz, Poland), anti-factor Xa assay kit (Sekisui Diagnostics, Burlington, MA, USA), mouse sP-selectin/CD62P, mouse thrombopoietin (R&D Systems, Inc., Minneapolis, MN, USA), D-dimer, PF4, βTG (Cloud-clone corp., Katy, TX, USA), and 6-keto-PGF1α ELISA kits (Cayman Chemicals, Ann Arbor, MI, USA), and collagen (Chrono-log, Havertown, PA, USA). 

### 4.2. Animals and Housing

The animals were obtained from the Centre of Experimental Medicine at the Medical University of Bialystok. The animals were bred in a 12-hour light/dark cycle, in a temperature- and humidity-controlled room, and were allowed to have ad-libitum access to sterilized water and standard chow in specific pathogen-free conditions. All of the procedures involving animals were approved by the Local Ethical Committee on Animal Testing (permits: 92/2012, 108/2015, 2/2018, and 60/2018), and were conducted by Directive 2010/63/EU of the European Parliament and the Council on the Protection of Animals, the ARRIVE guidelines, and the national laws. The animals were euthanized by exsanguination at the end of the experiments.

### 4.3. Experiment 1: The Number of Platelets and Their Aggregation up to 60 Min after a Single Injection of UFH and PS into Mice

**Figure 8:**
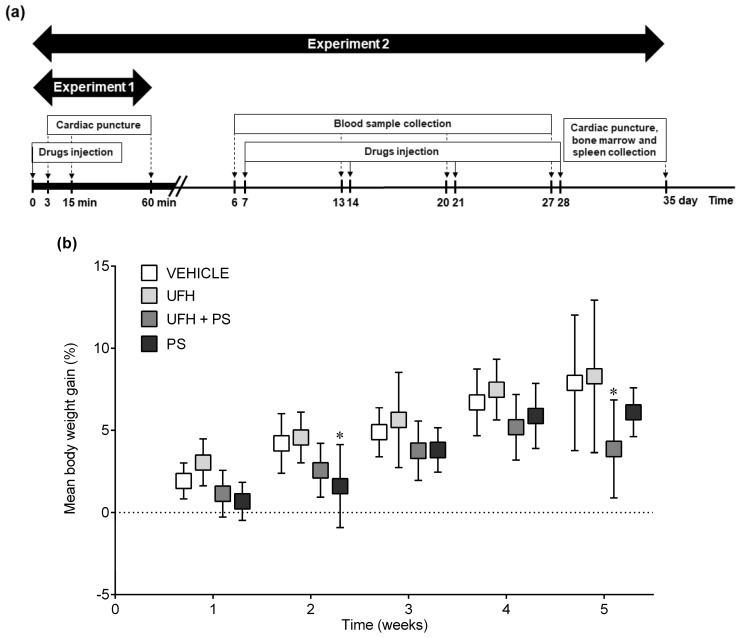
Schematic representation of (**a**) the study protocol, and (**b**) the mean body weight gain expressed as a percentage of the body weight before treatment with unfractionated heparin (UFH) and protamine sulfate (PS) in mice (*n* = 8) during the second experiment. The results are shown as mean ± standard deviation (SD).* *p* < 0.05 vs. vehicle, ANOVA with Fisher’s LSD post hoc test.

The blood samples were collected from the hearts each of 71 male BALB/c mice (21.1 ± 2.4 g), at five weeks old, after being anesthetized with a mixture of isoflurane and medical air (1.5–3% *v*/*v*), and were drawn into 3.13% trisodium citrate in a volume ratio of 9:1 at the end of the experiment. The blood cells were counted 3, 15, and 60 min following injection into the right femoral vein of the vehicle (phosphate buffered saline, 1 mL/kg), UFH (150 U/kg, 1 mL/kg), PS (1.5 mg/kg, 1 mL/kg), or both (Figure 8a), using the Animal Blood Counter (ABC Vet, Horiba ABX Sp. z o.o., Warsaw, Poland). At the same time, the points platelet aggregation was measured after the incubation of the whole blood (500 µL) and 0.9% NaCl solution (500 µL) for 20 min at 25 °C, and then for 15 min at 37 °C. The changes in impedance were registered for 6 min after the collagen addition (5 µg/mL) using a Chrono-log aggregometer (Chrono-log Corp., Havertown, PA, USA). The aggregation curve was described by the maximal extension, the slope of the platelet aggregation, lag phase, and the area under the curve. 

### 4.4. Experiment 2: The Number of Platelets and Their Aggregation, Bone Marrow Cytology, and Coagulation Parameters 35 Days after the Repeated (Once a Week) Injection of UFH and PS into Mice

PS (1.5 mg/kg, 1 mL/kg) and UFH (150 U/kg, 1 mL/kg) alone or in combination were injected once weekly into the tail veins of 32 male BALB/c mice (26.0 ± 1.7 g), eight weeks old, during five weeks. The vehicle-treated (phosphate buffered saline, 1 mL/kg) animals served as a control group. The blood was collected four times from each animal, anesthetized with a mixture of isoflurane and medical air (3% *v*/*v*) by puncture of the retro-orbital plexus on the day before the drug administration (Figure 8a). The blood samples were centrifuged at 8000 rpm at 22 °C for 5 min after 1-h of incubation at room temperature, and the serum was deep-frozen (−80 °C) until the further determination of TPO concentration by immunoassay (R&D Systems, Minneapolis, MN, USA). No deaths, drug-related clinical signs of toxicity, effects on food consumption, or visual changes were reported during the 35-day observation. We did not observe significant differences in the mean baseline body weights between the treatment groups. The mean body weight did not increase equally in all of the groups. We only noted a slight, but significant, decrease in body weight gain in the mice treated with PS and with UFH and PS in the second and fifth week of the experiment, respectively (Figure 8b). Blood was collected from the hearts under anesthesia (a mixture of isoflurane and medical air; 3% *v*/*v*) one week after the last drug dose administration. The platelet aggregation and blood cell count were measured according to the methods described above, with the collagen addition at a concentration of 5 µg/mL. The serum was separated and used for the determination of TPO, P-selectin (R&D Systems, Minneapolis, MN, USA), PF4, and the βTG concentration (Cloud-Clone Corp., Katy, TX, USA) in a microplate reader (Synergy HTX, BioTek, Winooski, VT, USA), according to the kit manufacturer’s directions. Sodium citrate anticoagulated blood samples were centrifuged at 3500× *g* at 4 °C for 10 min, and the plasma was deep-frozen (−80 °C) until further assays could be performed. The aPTT was automatically determined by an optical method (Coag Chrom 4000, Bio-Ksel, Grudziadz, Poland), adding routine laboratory reagents (Bio-Ksel, Grudziadz, Poland). The plasma concentration of D-dimer was measured by the ELISA technique, using a microplate reader (Synergy HTX, BioTek, Winooski, VT, USA) to monitor the changes in absorbance according to the kit manufacturer directions (Cloud-Clone Corp., Katy, TX, USA). Following the blood collection, the animals were euthanized, and the bone marrow from the femurs were collected immediately for cytological examination. The bone marrow smears were stained by the May–Grünwald–Giemsa method. The morphology was assessed by examination with a ×40 objective, and a 100-cell count for the bone marrow was performed with a ×100 objective. Any cell that did not fit the definition was counted with the category it most closely resembled. 

### 4.5. Experiment 3: The Number of Platelets and Their Aggregation up to 60 Min after a Single Injection of UFH and PS into Rats

**Figure 9:**
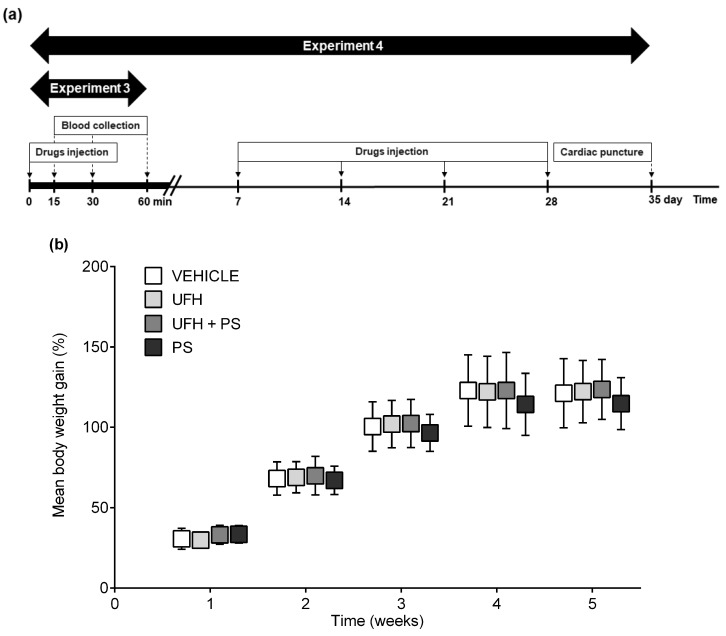
Schematic representation of (**a**) the study protocol and (**b**) the mean body weight gain expressed as a percentage of the body weight before treatment with unfractionated heparin (UFH) and protamine sulfate (PS) in rats (*n* = 9–10) during the fourth experiment. The results are shown as mean ± SD. ANOVA with Fisher’s LSD post-hoc test.

The blood samples were collected from the tail arteries of 32 male Wistar rats (177.7 ± 13.5 g), at seven weeks old, after being anesthetized by an intraperitoneal injection of pentobarbital (45 mg/kg), using K_2_EDTA as an anticoagulant. The blood cells were counted 15, 30, and 60 min following injection into the right femoral vein of the vehicle (phosphate buffered saline, 1 mL/kg), UFH (150 U/kg, 1 mL/kg), PS (1.5 mg/kg, 1 mL/kg), or both (Figure 9a), using the Animal Blood Counter (ABC Vet, Horiba ABX Sp. z o.o., Warsaw, Poland). The blood samples were collected from the heart and drawn into 3.13% trisodium citrate in a volume ratio of 9:1 at the end of the experiment. The platelet aggregation was measured according to the methods previously described, with the collagen addition at a concentration of 7.5 µg/mL.

### 4.6. Experiment 4: The Number of Platelets and Their Aggregation, Arterial Thrombosis, and Coagulation Parameters 35 Days after the Repeated (Once a Week) Injection of UFH and PS into Rats

PS (1.5 mg/kg, 1 mL/kg) and UFH (150 U/kg, 1 mL/kg), alone or in combination, were injected once a week into the tail veins of 38 male Wistar rats (130.5 ± 9.9 g), at five weeks old, after being anesthetized with a mixture of isoflurane and medical air (3% *v*/*v*; Figure 9a). The vehicle-treated (phosphate buffered saline, 1 mL/kg) animals served as a control group. No deaths, drug-related clinical signs of toxicity, effects on food consumption, or visual changes were observed in the study. We did not observe significant differences in the mean baseline body weights between the randomized treatment groups. The mean body weight of the control rats increased from 129.0 ± 9.6 g at baseline, to 283.6 ± 7.8 g during 35 days, and it was similar in the rats receiving drugs (Figure 9b). Arterial thrombosis was induced by the electrical stimulation (1 mA/10 min) of the common carotid artery during anesthesia with pentobarbital (45 mg/kg, i.p.), one week after the last drug administration, as described previously [30]. The formed thrombus was completely removed 45 min after thrombosis induction, air-dried at 37 °C, and weighed 24 h after the end of the experiment. The blood cell count and platelet aggregation were measured according to the methods described above, with the collagen addition at a concentration of 7.5 µg/mL. The sodium citrate anticoagulated blood samples were centrifuged at 3500× *g* at 4 °C for 20 min, and the plasma was deep-frozen (−80 °C) until further assays could be performed. The aPTT, PT, and fibrinogen concentrationd were automatically determined by an optical method (Coag Chrom 4000, Bio-Ksel, Grudziadz, Poland), adding routine laboratory reagents. The plasma concentrations of 6-keto-PGF1α (Cayman Chemicals, Ann Arbor, MI, USA) and D-dimer (Cloud-clone corp., Katy, TX, USA) were measured by the ELISA technique, using a microplate reader (Synergy HTX, BioTek, Winooski, VT, USA) to monitor the changes in absorbance according to the kit manufacturer directions.

### 4.7. Statistical Analysis

In the study, *n* refers to the number of animals in each experimental group. We chose a minimal number of animals to detect the differences between each group based on our experience as well as others’ experience using these procedures. The data are shown as the median, with the lower and upper limits, or mean ± SD. All of the data sets were tested for normality with the Shapiro–Wilk test. Multiple group comparisons were performed using the ANOVA with Dunn’s or Fisher’s LSD post-hoc tests, depending on whether the data have a normal or non-normal distribution. The results were analyzed and graphically presented using GraphPad Prism 8 (GraphPad Software, La Jolla, CA, USA). *P* values < 0.05 were considered significant.

## 5. Conclusions

In summary, we showed a clear direct antiplatelet effect of PS in vivo, which was present 15–60 min from PS administration, and absent when PS was administered once a week or together with UFH. Interestingly, PS administered chronically inhibited arterial thrombosis in rats. However, it is impossible to consider PS as an antithrombotic medicine, because of its serious adverse cardiovascular effects. PS dosing is usually based on clinical experience rather than evidence. The additional PS doses are still commonly administered for the neutralization of residual UFH, because of the phenomenon known as “heparin rebound”. This side reaction is associated with serious postoperative bleeding, and is explained by PS underdosing. Our in vivo study shows that for hemorrhage complications in patients during cardio-surgical procedures, the antiplatelet activity of excess PS could be responsible. Activated clotting time or aPTT tests used to monitor the anticoagulant activity of UFH should be interpreted carefully when PS is administered to restore coagulation, as these tests are insensitive to the antiplatelet effect of PS, and bleeding may still occur. Our results showed that PS may still exert an antithrombotic effect even one week after the last administration, which may increase the risk of bleeding, especially in patients having had previous contact with PS and taking anticoagulants drugs. Importantly, our conclusions support the results from two different species.

## Supplementary Materials

Supplementary materials can be found at https://www.mdpi.com/1660-3397/17/9/539/s1, Table S1: The percentage of cells of various categories in bone marrow of mice on the 35th day of the experiment, Table S2: Complete blood count in rats after UFH and PS administration alone or in combination during the one-hour observation and on the 35th day of the experiment, Table S3: Complete blood count in mice at 3, 15 and 60 min, and on the 35th day after UFH and PS administration alone or in combination.

## Abbreviations

anti-fXa	anti-factor Xa
aPTT	activated partial thromboplastin time
βTG	β-thromboglobulin
CPB	cardiopulmonary bypass
ELISA	enzyme-linked immunosorbent assay
GPIb	glycoprotein Ib
IgG	immunoglobulin G
NO	nitric oxide
NPH	neutral protamine Hagedorn insulin
PF4	platelet factor 4
PolyP	polyphosphates
PS	protamine sulfate
PT	prothrombin time
TPO	thrombopoietin
UFH	unfractionated heparin
vWF	von Willebrand factor
VEH	vehicle
