# Supplementary material for: The Inhibitory Effect of Protamine on Platelets is Attenuated by Heparin without Inducing Thrombocytopenia in Rodents
Source: Mar Drugs. 2019 Sep 17;17(9):539. doi: 10.3390/md17090539 (PMC6780366; doi:10.3390/md17090539)
Supplement: Supplementary file 1 [file marinedrugs-17-00539-s001.pdf]

# Supplementary Data

# The Inhibitory Effect of Protamine on Platelets is Attenuated by Heparin without Inducing Thrombocytopenia in Rodents

**Joanna Miklosz [1], Bartlomiej Kalaska [1], Kamil Kaminski [2], Malgorzata Rusak [3], Krzysztof Szczubialka [2], Maria Nowakowska [2], Dariusz Pawlak [1] and Andrzej Mogielnicki [1,]***

[1] Department of Pharmacodynamics, Medical University of Bialystok, 15-222 Bialystok, Poland
[2] Department of Physical Chemistry, Faculty of Chemistry, Jagiellonian University, 30-387 Krakow, Poland
[3] Department of Haematological Diagnostics, Medical University of Bialystok, 15-269 Bialystok, Poland
* Correspondence: andrzej.mogielnicki@umb.edu.pl; Tel.: +48-85-748-5889

**Table S1.** The percentage of cells of various categories in bone marrow of mice on the 35th day of the experiment.

| Parameter | VEH | UFH | UFH + PS | PS |
|---|---|---|---|---|
| **Erythroid (E)** | | | | |
| Proerythroblasts | 1.5 (0–3) | 1.5 (0–4) | 1.5 (0–6) | 0.5 (0–3) |
| Basophilic erythroblasts | 6.0 (3–7) | 4.5 (3–9) | **3.0 (0–4)**** | 4.0 (1–7) |
| Polychromatic erythroblasts | 9.0 (7–15) | 7.5 (1–14) | 8.5 (4–11) | **6.5 (4–11)*** |
| Oxyphilic erythroblasts | 6.5 (4–10) | 5.0 (1–10) | 5.5 (5–9) | 6.5 (3–9) |
| **Total of erythroid cells** | 21.5 (18–30) | 19.0 (7–29) | 17.5 (9–24) | **15.5 (12–24)*** |
| **Myeloid (M)** | | | | |
| Myeloblasts | 0.0 (0–2) | 0.0 (0–1) | 0.0 (0–2) | 0.0 (0–1) |
| Promyelocytes | 5.5 (3–11) | **3.5 (1–5)*** | 5.5 (3–10) | **3.5 (2–7)*** |
| Myelocytes | 21.5 (17–25) | 21.5 (19–24) | 20.5 (19–26) | 21.5 (20–26) |
| Metamyelocytes | 16.0 (13–18) | 17.0 (15–23) | **18.0 (15–22)*** | **18.0 (15–25)**** |
| Band | 19.0 (10–23) | 20.0 (13–26) | 19.0 (18–21) | 20.5 (16–23) |
| Neutrophils | 14.0 (6–22) | 13.5 (9–25) | 15.0 (11–19) | 16.0 (8–31) |
| Eosinophils | - | - | 0.0 (0–1) | - |
| Basophils | - | - | - | - |
| **Total of myeloid cells** | 75.5 (65–83) | 75.5 (68–92) | 79.5 (74–88) | 79.5 (73–90) |
| Lymphocytes | 4.0 (3–7) | **2.0 (1–8)*** | **2.0 (1–3)***** | 3.0 (0–4) |
| Monocytes | 0.0 (0–1) | 0.5 (0–2) | 0.0 (0–1) | 0.5 (0–1) |
| **M/E ratio** | 3:1 | 4:1 | 4:1 | 5:1 |

Abbreviations: VEH: vehicle, UFH: unfractionated heparin, PS: protamine sulfate. *$p < 0.05$, **$p < 0.01$, ***$p < 0.001$ vs vehicle, Kruskal-Wallis ANOVA with Dunn's post hoc test. Results are shown as median with range; $n = 8$.

**Table S2.** Complete blood count in rats after UFH and PS administration alone or in combination during the one-hour observation and on the 35th day of the experiment.

| Parameter | T | VEH | UFH 150 U/kg | UFH 150 U/kg + PS 1.5 mg/kg | PS 1.5 mg/kg |
|---|---|---|---|---|---|
| WBC ($10^3/mm^3$) | **15 min** | 4.8 (1.9–6.8) | 3.7 (3.6–7.8) | 4.0 (3.3–6.5) | 3.6(2.2–5.5) |
| | **30 min** | 4.3 (2.0–6.4) | 3.5 (3.2–4.1) | 4.1 (1.1–10.1) | 3.8 (3.2–5.1) |
| | **60 min** | 2.2 (1.6–6.4) | **2.9 (2.1–3.3)^** | 3.1 (2.4–9.8) | **2.3 (2.1–4.3)**# |
| | **35 day** | 3.2 (2.3–3.9) | 3.0 (2.4–4.1) | 2.7 (1.8–3.6) | 3.1 (1.9–6.1) |
| RBC ($10^6/mm^3$) | **15 min** | 7.1 (6.6–8.1) | 7.4 (6.7–8.1) | 7.1 (6.4–7.9) | 7.2 (6.9–8.3) |
| | **30 min** | 7.1 (6.9–8.1) | 7.2 (7.0–7.9) | 7.2 (6.7–8.1) | 7.1 (6.8–8.3) |
| | **60 min** | 6.5 (5.8–7.9) | **7.0 (6.2–7.9)^** | **6.6 (6.3–7.9)^** ## | **6.6 (5.7–7.0)^^** ## |
| | **35 day** | 8.8 (7.7–9.2) | 8.5 (8.2–9.3) | 8.6 (7.9–9.4) | 8.8 (8.5–9.2) |
| HGB (g/dl) | **15 min** | 14.6 (13.4–14.9) | 14.0 (13.2–14.8) | 14.1 (13.5–15.4) | 14.0 (13.8–15.6) |
| | **30 min** | 14.8 (13.2–14.9) | 13.7 (12.7–13.9) | 13.7 (13.4–15.7) | 14.0 (13.3–15.4) |
| | **60 min** | 12.5 (11.3–15.3) | 13.1 (12.9–13.9) | **12.8 (12.2–15.4)^^** # | **12.7 (10.9–14.1)^^** # |
| | **35 day** | 15.9 (14.5–16.8) | 15.9 (15.2–16.5) | 15.8 (14.6–17.1) | 16.6 (15.2–17.2) |
| HCT (%) | **15 min** | 44.2 (38.4–47.0) | 43.5 (41.7–46.5) | 44.2 (41.1–46.2) | 43.8 (43.2–49.4) |
| | **30 min** | 45.2 (41.5–46.8) | 42.5 (39.9–43.5) | 43.2 (41.6–48.1) | 43.93 (41.7–49.2) |
| | **60 min** | 38.7 (35.4–46.9) | **41.3 (40.8–43.8)^** | **39.6 (37.9–46.6)^** # | **39.7 (37.4–42.8)^^** # |
| | **35 day** | 48.7 (44.3–50.9) | 48.1 (45.6–51.3) | 48.0 (43.8–51.6) | 48.9 (46.9–52.5) |
| MCV ($\mu m^3$) | **15 min** | 60.0 (58–63) | 59.0 (57–60) | 60.0 (59–62) | 60.5 (59–63) |
| | **30 min** | 60.0 (58–64) | 59.0 (57–60) | 60.0 (59–62) | 60.5 (59–63) |
| | **60 min** | 59.0 (57–)64 | 59.0 (57–60) | 60.0 (59–62) | 60.0 (59–63) |
| | **35 day** | 56.0 (54–58) | 56.0 (55–58) | 56.0 (55–58) | 55.0 (53–58) |
| MCH (pg) | **15 min** | 20.0 (18.5–21.0) | **18.8 (18.2–19.0)*** | 19.6 (18.9–20.4) | 19.3 (18.7–20.1) |
| | **30 min** | 19.1 (18.3–21.0) | 18.6 (18.1–19.3) | **19.3 (18.8–20.3)^** | 19.3 (18.5–20.0) |
| | **60 min** | 18.9 (18.3–21.6) | 18.5 (18.0–19.2) | **19.3 (18.7–20.2)^** | 19.3 (18.8–20.3) |
| | **35 day** | 18.5 (17.6–19.0) | 18.5 (17.7–19.2) | 18.6 (18.2–19.0) | 18.3 (17.3–19.4) |
| MCHC (g/dl) | **15 min** | 32.9 (31.2–34.9) | 31.7 (31.4–32.2) | 32.2 (31.4–33.3) | 31.9 (31.5–32.3) |
| | **30 min** | 31.7 (31.4–33.0) | 31.9 (31.5–32.7) | **32.0 (31.6–32.7)^** | 31.9 (31.2–32.5) |
| | **60 min** | 31.9 (31.7–35.4) | 31.8 (31.3–32.6) | 32.1 (31.5–33.1) | 32.2 (31.4–33.0) |
| | **35 day** | 33.0 (32.1–33.6) | 32.9 (32.2–33.7) | 33.1 (32.8–33.7) | 32.8 (32.3–34.0) |

Abbreviations: WBC: white blood cells, RBC: red blood cells, HGB: hemoglobin, HCT: haematocrit, MCV: mean corpuscular volume, MCH: mean corpuscular haemoglobin, MCHC: mean corpuscular haemoglobin concentration, T: time, UFH: unfractionated heparin, PS: protamine sulfate. ***** $p < 0.05$ vs vehicle at the corresponding time point, Kruskal-Wallis ANOVA with Dunn's post hoc test; **^** $p < 0.05$, **^^** $p < 0.001$ vs 15 min within same group; # $p < 0.05$, ## $p < 0.01$ vs 30 min within same group, Friedman ANOVA with Dunn's post hoc test. Results are shown as median with range; $n$ = 5–10.

**Table S3.** Complete blood count in mice at 3, 15 and 60 min, and on the 35th day after UFH and PS administration alone or in combination.

| Parameter | T | VEH | UFH 150 U/kg | UFH 150 U/kg + PS 1.5 mg/kg | PS 1.5 mg/kg |
|---|---|---|---|---|---|
| **WBC ($10^3$/mm$^3$)** | **3 min** | 0.6 (0.4–1.0) | 0.8 (0.2–1.3) | 0.5 (0.2–0.6) | 0.3 (0.2–1.7) |
| | **15 min** | | **0.4 (0.2–0.5)*** | 0.5 (0.2–0.8) | 1.5 (0.4–6.4) |
| | **60 min** | 1.5 (1.4–2.2) | 1.8 (1.2–2.7) | 1.7 (1.2–2.3) | 1.7 (1.3–2.9) |
| | **35 day** | 1.8 (0.8–4.5) | 1.4 (0.9–2.2) | 1.6 (0.7–5.5) | 1.4 (0.4–2.1) |
| **RBC ($10^6$/mm$^3$)** | **3 min** | 7.4 (7.1–7.6) | **7.1 (5.9–7.9)*** | **7.0 (6.6–7.1)**** | 7.2 (6.2–7.8) |
| | **15 min** | | 7.1 (6.6–8.1) | **7.0 (6.5–7.4)*** | 7.4 (6.6–7.8) |
| | **60 min** | 7.2 (6.9–8.1) | 7.6 (6.9–7.9) | 7.6 (7.5–7.6) | 7.6 (6.9–7.9) |
| | **35 day** | 4.3 (4.0–4.6) | 4.2 (3.9–4.5) | 4.2 (4.0–4.5) | 4.2 (4.0–4.3) |
| **HGB (g/dl)** | **3 min** | 12.7 (12.4–13.0) | 12.3 (10.6–14.1) | **11.8 (11.2–12.1)**** | 12.1 (10.5–13.4) |
| | **15 min** | | 12.2 (11.0–14.1) | **11.9 (11.1–12.8)*** | 12.4 (11.3–13.5) |
| | **60 min** | 11.9 (11.4–13.1) | 12.2 (10.8–13.2) | 12.4 (12.1–12.9) | 12.4 (11.6–13.0) |
| | **35 day** | 12.7 (12.3–13.1) | 12.4 (11.0–12.8) | **12.0 (11.5–13.2)*** | 12.3 (11.7–12.8) |
| **HCT (%)** | **3 min** | 41.4 (39.5–41.9) | 40.0 (33.9–44.1) | **38.6 (38.1–40.9)*** | 40.1 (34.4–43.3) |
| | **15 min** | | 40.2 (36.3–44.8) | 39.1 (36.8–42.2) | 41.8 (36.5–44.3) |
| | **60 min** | 37.9 (36.2–43.6) | 39.7 (35.2–43.2) | 40.7 (40.1–41.5) | 40.1 (36.9–42.3) |
| | **35 day** | 33.0 (31.3–35.7) | 32.6 (30.5–35.0) | 32.6 (31.1–34.8) | 32.1 (31.0–33.0) |
| **MCV (μm$^3$)** | **3 min** | 55.0 (54–56) | **57.0 (54–58)**** | **57.0 (55–58)*** | 56.0 (55–57) |
| | **15 min** | | 56.0 (55–58) | **56.5 (55–57)*** | **56.0 (55–57)*** |
| | **60 min** | 53.0 (53.0–54.0) | 52.0 (51.0–55.0) | 54.0 (53.0–55.0) | 53.5 (52.0–54.0) |
| | **35 day** | 78.0 (76–79) | 78.0 (77–78) | 77.0 (76–78) | 77.0 (76–78) |
| **MCH (pg)** | **3 min** | 17.4 (16.9–17.5) | 17.6 (17.1–17.9) | 16.9 (16.1–17.7) | 17.2 (16.8–17.7) |
| | **15 min** | | 17.2 (16.8–17.8) | 17.1 (16.4–17.8) | 17.1 (16.5–17.5) |
| | **60 min** | 16.6 (16.2–16.6) | 16.6 (15.8–16.8) | 16.4 (16.1–17.0) | 16.4 (16.2–16.9) |
| | **35 day** | 29.4 (28.2–31.1) | 28.3 (28.1–29.5) | 29.0 (27.4–29.4) | 29.8 (28.8–30.2) |
| **MCHC (g/dl)** | **3 min** | 31.2 (30.6–31.9) | 30.7 (30.1–32.4) | **29.2 (29.1–31.6)*** | 30.7 (29.6–31.7) |
| | **15 min** | | 30.8 (29.3–31.9) | **30.4 (29.0–31.9)*** | 30.8 (28.8–31.2) |
| | **60 min** | 31.4 (30.0–31.5) | 30.9 (30.6–31.7) | 30.6 (29.8–31.0) | 30.9 (30.7–31.5) |
| | **35 day** | 38.2 (36.6–39.4) | **36.7 (36.0–38.0)*** | 37.5 (35.8–38.0) | 38.8 (37.0–39.2) |

Abbreviations: WBC: white blood cells, RBC: red blood cells, HGB: hemoglobin, HCT: haematocrit, MCV: mean corpuscular volume, MCH: mean corpuscular haemoglobin, MCHC: mean corpuscular haemoglobin concentration, T: time, UFH: unfractionated heparin, PS: protamine sulfate. *$p < 0.05$, **$p < 0.01$ vs vehicle, Kruskal-Wallis ANOVA with Dunn's post hoc test. Results are shown as median with range; $n$ = 5–8.
